# Confocal imaging of single BaTiO_3_ nanoparticles by two-photon photothermal microscopy

**DOI:** 10.1038/s41598-017-01548-z

**Published:** 2017-05-10

**Authors:** M. M. Bijeesh, P. K. Shakhi, S. Arunkarthick, Geetha K. Varier, P. Nandakumar

**Affiliations:** Department of Physics, Birla Institute of Technology and Science, Pilani K. K. Birla Goa Campus, Goa, 403726 India

## Abstract

We report on the development of a nonlinear optical microscopic technique based on two-photon absorption induced photothermal effect capable of detecting individual nonfluorescent nanoparticles with high sensitivity. The method which is inherently confocal makes use of near infrared excitation at high repetition rates and would be of interest in deep tissue imaging. We demonstrate the applicability of the technique by imaging single BaTiO_3_ nanoparticles, a potential biomolecular label having high photostability, in a scattering environment at fast time scales with a pixel dwell time of 80 μs.

## Introduction

Confocal fluorescence microscopy is one of the most successful and widely used imaging tools in Biology^1–3^. High sensitivity, spatial resolution and optical sectioning capability make it an ideal tool in the study of biological systems such as membranes, tissues and cells^1, 2^. However the success of all fluorescence microscopies relies heavily on the availability of suitable fluorophores that can be used to stain the region of interest. A major issue faced by most biologists employing fluorescence microscopy is the phototoxicity and photobleaching of available fluorophores^2^. While phototoxicity of the fluorophore is inherently harmful to the biological system under observation, photobleaching hinders the use of the technique in studies that require large observation time. This limitation of fluorescence microscopy has stimulated an intense research effort on developing new labels and new imaging techniques^4–8^. Though these efforts have partly succeeded in developing new photostable labels such as semiconductor quantum dots, their usage as labels is often limited due to irregular photoblinking^4, 5^. In parallel different label free imaging techniques based on Raman scattering such as coherent anti-stokes Raman scattering microscopy^6^ and stimulated Raman scattering microscopy^7, 8^ has been developed in recent years to altogether circumvent the necessity for dye labeling in imaging. Though there is a continuing effort to improve their functionality, these techniques are limited to specific systems and have not reached itself to standard biology laboratories primarily due to the complexities in the setup^9^.

It is in this context that absorption based detection of non-luminescent nanosize particles through photothermal techniques become promising. Photothermal microscopy is a pump-probe detection technique where one detects the pump induced temperature variation around a nanoparticle using a probe beam. Detection of gold nanoparticles using photothermal microscopy has been reported recently^10–14^. Berciaud *et al*. have succeeded in the detection of gold nanoparticle of size as small as 1.4 nm with photothermal heterodyne imaging^15^. This method of detection of gold nanoparticles is free from photobleaching and high signal to noise ratio (SNR) images can be acquired at fast timescales suitable for biological imaging^16^. Photothermal effects in gold nanorods has been successfully employed in cancer cell imaging and selective photothermal cancer therapy in *in vitro* conditions^17, 18^. Applicability of photothermal microscopy using gold nanorods in biomolecular imaging and the techniques potential as noninvasive alternative to surgery in cancer therapy has been further demonstrated by Tong *et al*.^19, 20^. These studies show that photothermal microscopy is particularly promising from the point of view of biomedical applications. However photothermal microscopy in its present form makes use of linear optical absorption and is not inherently confocal. Further, the usage of gold nanoparticles as label necessitates the use of visible light having wavelength around 500 nm as pump beam. This wavelength is not ideal for biomolecular imaging, especially in experiments that involve deep tissue imaging, due to the absorption in biological tissues which ranges from 350 nm to 650 nm. These drawbacks of conventional photothermal microscopy advocate the use of two-photon excitation induced thermal effects as a contrast mechanism in biological imaging. Optical absorption in two-photon excitation microscopy is restricted to the focal plane of the microscope. Hence two-photon excitation microscopy is inherently confocal and would allow deeper penetration. Advantages of two-photon excitation fluorescence microscopy in three dimensional and deep tissue imaging have already been demonstrated^21–23^. Recently, Abeyasinghe *et al*. successfully employed two-photon excited fluorescence near-field scanning optical microscopy to image and study monolayer protected gold quantum dot composed of 25 gold atoms^24^. Applicability of two-photon excited photothermal microscopy in direct imaging of micrometer sized red blood cells containing heme proteins has been demonstrated recently^25, 26^. However many of the components of a complex biological system absorbs infrared radiation through two-photon absorption resulting in similar thermal profiles. In order to avoid this one may have to use very low pump powers limiting the applicability of the above technique to specific systems. One may overcome these difficulties by identifying and employing an appropriate nonlinear optical label possessing good thermal relaxation properties. Further this approach of using a photostable nanoparticle label will be particularly useful in live cell experiments that involve tracking of bimolecular transport.

In this work we report on the development of a two-photon photothermal microscope capable of detecting highly photostable and biocompatible barium titanate (BaTiO_3_) nanoparticle with high SNR. We make use of the large third order nonlinear optical susceptibility of BaTiO_3_ nanoparticles to excite them by means of two-photon absorption using a near infrared wavelength beam whose energy is half the energy difference between the transition levels. BaTiO_3_ being weakly fluorescent, the two-photon absorption phenomena creates a temperature profile around the particle by a nonradiative relaxation process. The corresponding refractive index variation is detected using a nonresonant probe beam. With this technique we are able to detect BaTiO_3_ nanoparticles in the size range of 20–100 nm at fast time scales with 80 µs pixel dwell time. The technique has the potential to be a viable alternative to confocal fluorescence microscopy. We demonstrate this capability by imaging BaTiO_3_ nanoparticles internalized HeLa cells. A 120 femtosecond (fs) laser pulse having only picojoules of energy at repetition rate of 76 MHz is used as the heating beam in this pump-probe microscopy. The applications of the two-photon photothermal microscope developed here are not limited to biological imaging, the microscope can be used as a potential noninvasive technique to detect and characterize nanosize objects in general.

## Results and Discussion

Two-photon absorption involves transition of a system from the ground state to a higher lying state by the simultaneous absorption of two photons from an incident radiation field. The rate of two-photon absorption is proportional to the square of the instantaneous intensity *I* and the optical loss due to absorption is described by the differential equation^27^

1 $$\frac{dI}{dz}=-\alpha I-\beta {I}^{2}$$

Where *α* is the linear absorption coefficient which in this case arise only from the impurities present, if any. The two-photon absorption coefficient *β* in this expression is a macroscopic parameter characterizing the material and is related to the imaginary part of the third order nonlinear optical susceptibility *x* ^(3)^ by the relation

2 $$\beta =\frac{3\pi }{{\varepsilon }_{0}{n}^{2}c\lambda }\text{Im}\,[{\chi }_{xxxx}^{3}(-\omega ;\omega ,\omega ,-\omega )]$$

Where *ε*_0_ is permittivity of free space, *n* is refractive index of the medium, *c* is speed of light, *λ* is wavelength of light and *ω* = 2*π*/*λ*. If *N* is the number density of molecules involved in the interaction, the transition rate due to the two-photon absorption process, *R* = *σ*_2_ *I* ^2^/*ħω* where *σ*_2_ = *ħωβ/N* is defined as the two-photon absorption cross section^27^. For a nonfluorescent material we may assume that most of the absorbed energy due to two-photon excitation is converted into heat and the nanoparticle would act as a point source of heat. It has been shown that gold nanorods having absorption in the near IR wavelength region efficiently converts absorbed light to heat and this property has been used in photothermal therapy^28^.

If the pump beam is modulated sinusoidally at a frequency Ω, then the absorbed power will vary as *P*_0_[1+cos(Ω*t*)] where the average absorbed power *P*_0_ is proportional to the two-photon absorption cross section and the square of the pump power, *P*_*pump*_. The dissipation of this energy into the medium will cause a temperature profile, ${\rm{\Delta }}T(r,t)\propto \frac{\,{\sigma }_{2}{P}_{Pump}^{2}}{4\pi \kappa r}[1+\,\cos ({\rm{\Omega }}t-\frac{{\rm{r}}}{{r}_{th}}){e}^{-\frac{r}{{r}_{th}}}]$ where *r* is the distance from the particle, ${r}_{th}=\sqrt{\frac{2\kappa }{{\rm{\Omega }}C}}$ is a characteristic length for heat diffusion, *κ* being the thermal conductivity of the surrounding medium and *C* its heat capacity. This temperature profile will lead to a time varying refractive index profile ${\rm{\Delta }}n(r,t)={\rm{\Delta }}T(r,t)\frac{\partial n}{\partial t}$ in the medium. A nonresonant probe beam interacting with this refractive index profile will result in a scattered field which can be used to map the thermal profile of the nanoparticle. In the experimental geometry employed, part of the incident probe field is reflected from sample-coverslip interface. This reflected probe field *E*_*R*_ interferes with the back scattered probe beam *E*_*s*_. The back scattered probe field is detected through its beatnote at the modulation frequency Ω using a lock-in amplifier. Berciaud *et al*.^29^ has carried out a detailed analysis of the polarization variations arising from local susceptibility fluctuations using the theory of light scattering^30^. As per this model the two-photon photothermal signal measured would be proportional to *P*_*Phi*_ where

3 $${P}_{phi}=n\frac{\partial n}{\partial t}\frac{f({\rm{\Omega }})}{C{\lambda }_{probe}^{2}{w}_{0}}{\sigma }_{2}{P}_{Pump}^{2}{P}_{Probe}$$

where *P*_*pump*_ and *P*_*probe*_ are power of pump beam and probe beam respectively. *w*_0_ is the probe beam focal radius and *λ*_*probe*_ is the wavelength of the probe beam. Here the size of the BaTiO_3_ nanoparticle is much smaller than the wavelength of the incident probe beam and hence the angular distribution of the scattered light is symmetric with respect to the focal plane. Since the spatial extension of the induced susceptibility profile is of microscopic dimensions we may assume that the frequency dependence of the signal *f* (Ω) can be approximated to 1/Ω at high pump modulation frequencies.

Two-photon excited laser scanning photothermal microscopy has several advantages in biological imaging over and above conventional photothermal microscopy. Unlike conventional photothermal microscopy, the technique is inherently confocal and would allow for three dimensional sectioning of the sample. Because there is no absorption in the out of focus areas, it assures more penetration into the specimen and thus allows deep tissue imaging. Further since near infrared wavelengths are used in the experiment there will be less scattering and thus less loss. A variety of ferroelectric materials like BaTiO_3_ and SrTiO_3_ have shown good nonlinear optical properties. BaTiO_3_ nanoparticles, used as a potential label in these experiments, exhibit large two-photon absorption cross-section near infrared region and is a biocompatible material that can be easily conjugated with biomolecules of interest^31–33^. Further, as an added advantage, BaTiO_3_ is low cost, and large variety of preparation techniques are available for in-house synthesis of the nanoparticles^34, 35^. The suitability of BaTiO_3_ nanoparticles in biological imaging has already been demonstrated and is used as a probe in the second harmonic generation (SHG) microscopy^36^. Hsieh *et al*. demonstrated harmonic holographic microscopy with BaTiO_3_ as second harmonic radiation imaging probes for high resolution 3D imaging of mammalian cells^33^. Culic-Viskota *et al*. successfully employed BaTiO_3_ as SHG nano probe in the *in vivo* imaging of zebrafish embryos^37^. However we may note that nanoparticles with cubic crystallization are not suitable for second harmonic generation microscopy because of the inversion symmetry.

**Figure 1:**
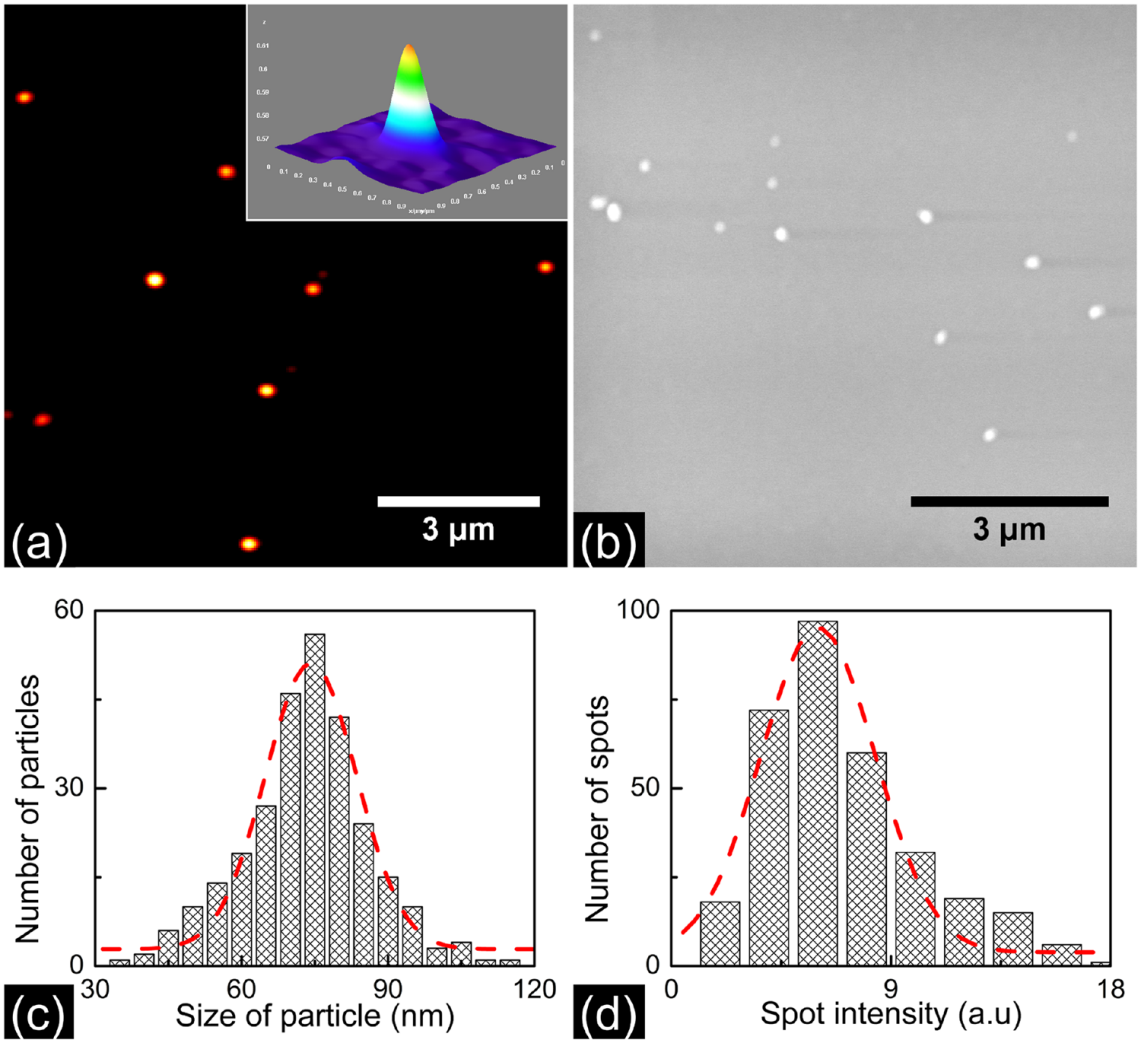
(**a**) Two-photon photothermal image of the 70 nm BaTiO_3_ nanoparticles spin coated on a coverslip at 4000 rpm for 30 s. The images are taken with 6 mW pump power and 0.8 mW probe power at the sample. The inset shows the pseudo 3D image of single BaTiO_3_ corresponding to the thermal profile generated. The SNR in the images shown is 47 (**b**) Scanning electron microscopy image of BaTiO_3_ having an average size of 70 nm prepared same as above. The figure shows single isolated BaTiO_3_ nanoparticles on the coverslip. (**c**) Particle size distribution histogram of BaTiO_3_ nanoparticle measured from the SEM images. The dotted line shows a Gaussian curve fit and the average particle size is found to be 70 nm and size distribution is (13 ± 2)%. (**d**) Two-photon photothermal intensity distribution histogram of BaTiO_3_ nanoparticles. The percentage distribution in intensity is (41 ± 5)%. Since the two-photon photothermal intensity scale with volume of the particle the intensity distribution is expected to be three times that of the particle size distribution.

**Figure 2:**
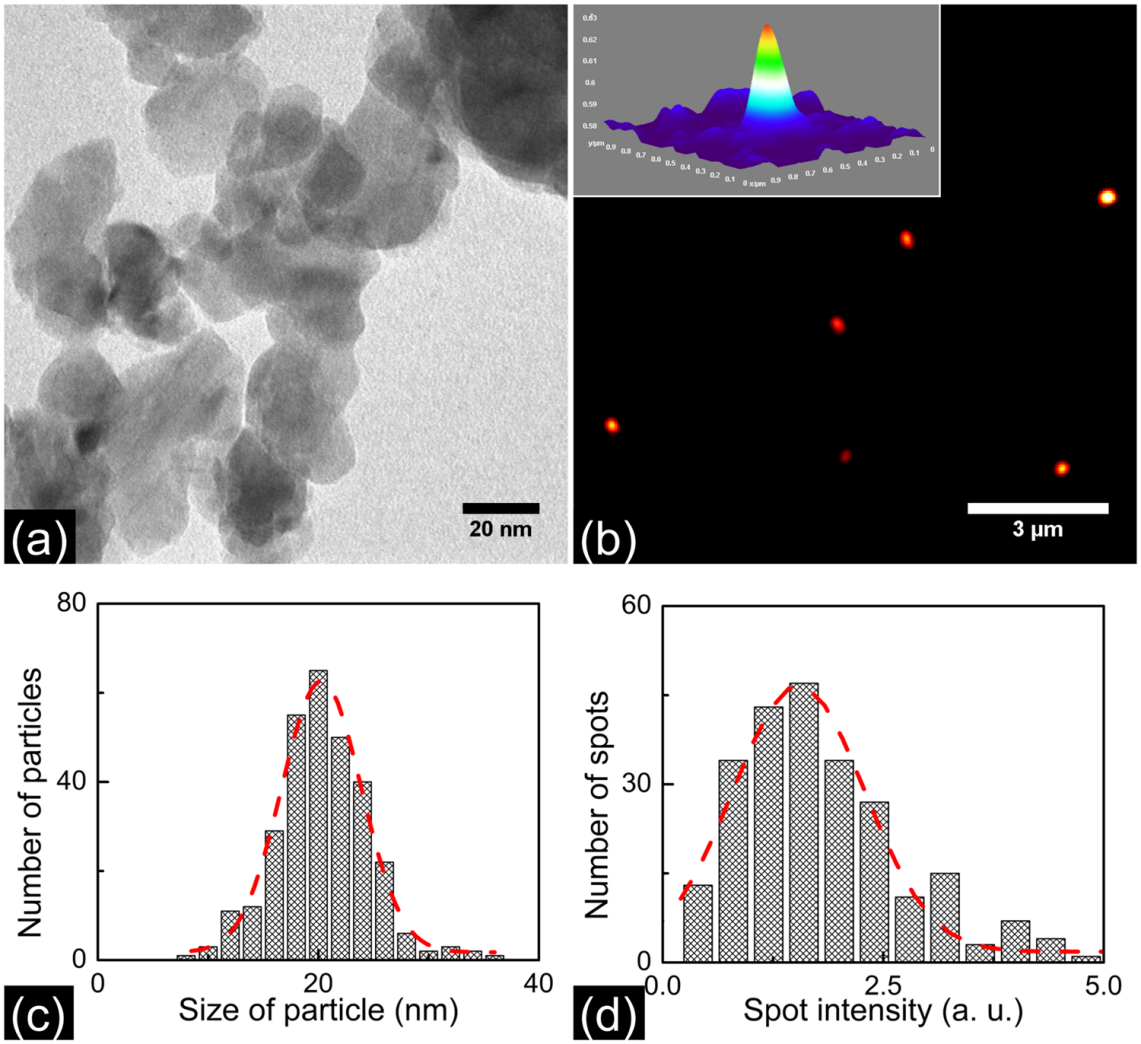
(**a**) TEM image of BaTiO_3_ nanoparticles prepared by sol-gel technique. (**b**) Photothermal image of 20 nm BaTiO_3_ nanoparticles spin coated on a coverslip at 4000 rpm for 30 s. The pump power and probe power used are 8 mW and 0.8 mW respectively. Inset shows pseudo 3D image of single 20 nm BaTiO_3_ nanoparticle. The SNR in the images shown is 19. (**c**) Particle size distribution of BaTiO_3_ nanoparticle measured from the TEM images. The dotted line shows a Gaussian curve fit. The average size of BaTiO_3_ nanoparticles is 20 nm. The percentage distribution in size is found to be (18 ± 2)%. (**d**) Two-photon photothermal intensity histogram of BaTiO_3_ nanoparticles. The percentage distribution in intensity is found to be (49 ± 6)%.

Figure 1(a) shows the two-photon photothermal image of 70 nm BaTiO_3_ nanoparticles acquired using the microscope as described in the methods section. The inset depicts the pseudo 3D image of one of the 8 particles seen in the figure. The images are acquired using pump pulses having energy of 80 picojoules (6 mW of average power). 0.8 mW of probe power is employed. A 500 × 500 pixel image is acquired in 20 s corresponding to a pixel integration time of 80 μs. The SNR in the images shown is 47. Here SNR is calculated by taking the ratio of the two-photon photothermal signal peak intensity in the image by standard deviation in the background. While taking the image it was verified that the signal disappears when either of the beams is blocked thereby making sure that no spurious scattered image is detected. In order to make sure that we are imaging single nanoparticles we compared the photothermal images with the scanning electron microscopy (SEM) images of an identically prepared sample. Figure 1(b) depicts the SEM image of a sample containing 70 nm BaTiO_3_ nanoparticles, prepared with the same spin speed as that employed for acquiring the photothermal images. The figure shows that well isolated single nanosize particles are formed on the coverslip at the 4000 rpm spin speed employed for preparing the sample. Figure 1(c) depict the size distribution of the nanoparticles as determined from the analysis of SEM images. The average particle size determined from the particle size distribution curve is 70 nm. The percentage distribution in size is found to be (13 ± 2)%. In order to further confirm that we are indeed detecting single nanoparticles, we compared the intensity distribution of the particles in the image to that of the size distribution of the synthesized particles. Since the photothermal intensity should scale with the volume of the particles, the corresponding photothermal intensity distribution is expected to be three times that of the particle size distribution. Figure 1(d) shows the two-photon photothermal intensity histogram of 70 nm BaTiO_3_ particle. The percentage distribution in intensity is (41 ± 5)% in agreement with expected value. We further repeated the experiment with smaller nanoparticles synthesized in the laboratory. Figure 2(a) shows the transmission electron microscopy (TEM) image of these BaTiO_3_ nanoparticles. In Fig. 2(b) we show the two-photon photothermal image of a thin film of BaTiO_3_ nanoparticles having an average size of 20 nm spin coated on a coverslip at a spin speed of 4000 rpm. The inset shows the pseudo 3D image of a single BaTiO_3_ nanoparticle corresponding to the thermal profile generated. The SNR in the images shown is 19. In Fig. 2(c) we depict the particle size distribution as determined from the analysis of the TEM images. The average size of BaTiO_3_ nanoparticles is 20 nm. The percentage distribution in size is found to be (18 ± 2)%. Figure 2(d) shows the two-photon photothermal intensity histogram. As expected the percentage distribution in intensity is found to be (49 ± 6)%.

**Figure 3:**
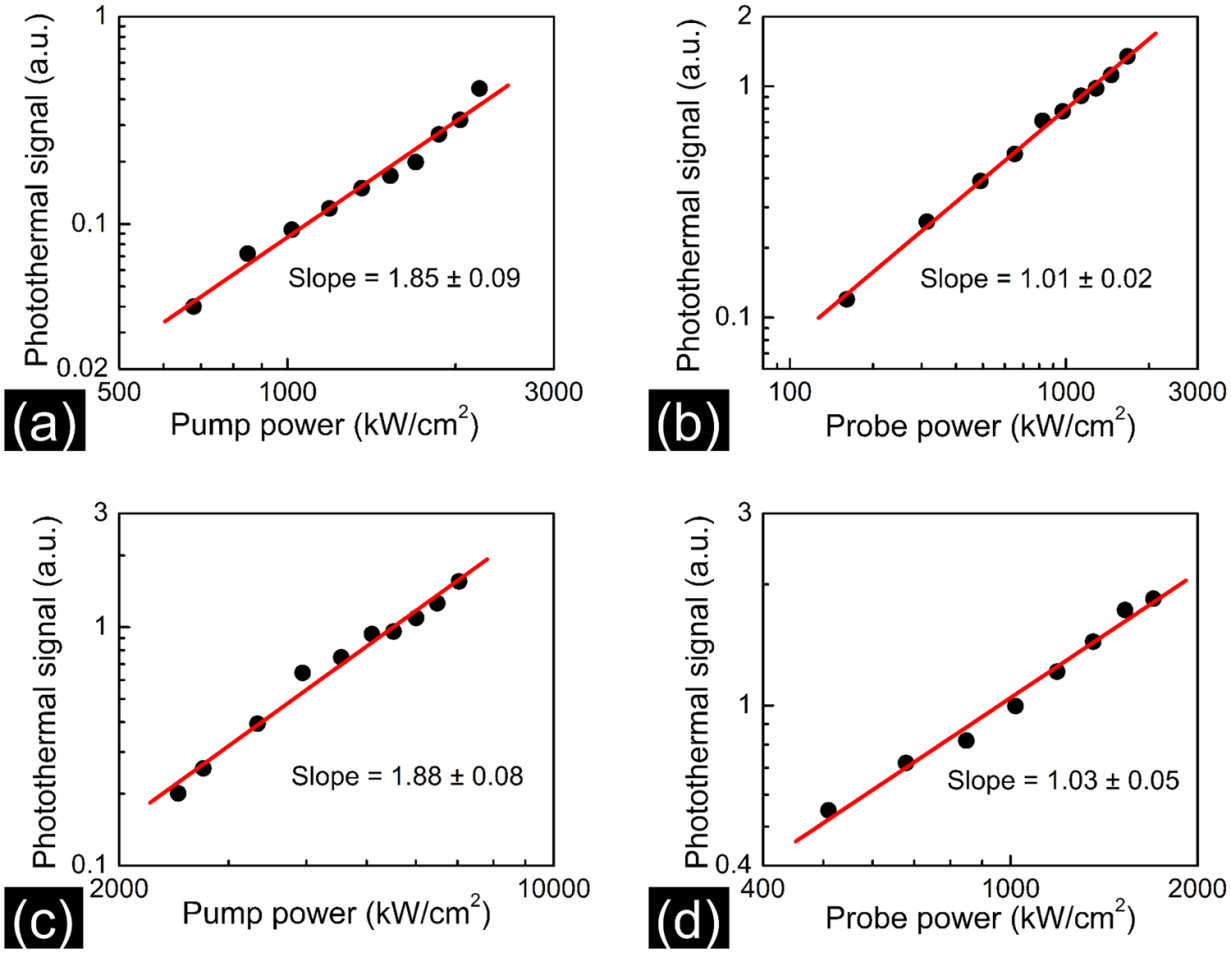
(**a**) pump and (**b**) probe power dependence of two-photon photothermal signal measured for 70 nm BaTiO_3_ nanoparticle. The probe power employed in (**a**) is 0.8 mW and the pump power employed in (**b**) is 6 mW. (**c**,**d**) shows the pump and probe power dependence of two-photon photothermal signal measured for 20 nm BaTiO_3_ nanoparticle respectively. The probe power employed in (**c**) is 0.8 mW and the pump power employed in (**d**) is 8 mW The signal varies quadratically with the pump power and linearly with the probe power.

In order to verify the power dependence predicted by equation (3) we focused the pump and probe beams on a single nanoparticle and looked at the photothermal intensity as function of incident power. Figure 3(a,b) shows the pump and probe power dependence of two-photon photothermal signal measured for 70 nm BaTiO_3_ nanoparticle. The best fit line to the data in Fig. 3(a) has a slope of 1.85 ± 0.09 which is close to the value of 2 predicted by equation (3). Figure 3(b) shows that the photothermal signal intensity depends linearly on probe power. Figure 3(c,d) shows the pump and probe power dependence of two-photon photothermal signal measured for 20 nm BaTiO_3_ nanoparticle respectively. Similar pump and probe power dependence is observed for both sets of particles.

**Figure 4:**
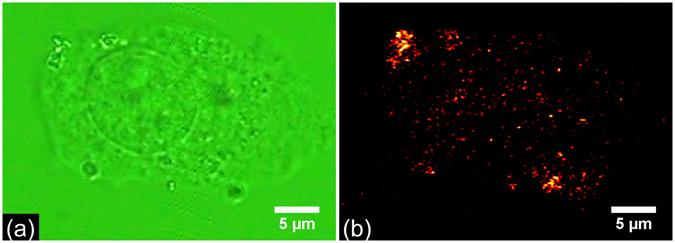
(**a**) Wide field image of the HeLa cell with PLL coated 20 nm BaTiO_3_ nanoparticles. (**b**) Corresponding 3D image of HeLa cells made from different axial sections of 2 μm interval taken with two-photon photothermal microscope. Pump and probe powers used are 3 mW and 0.8 mW respectively at the sample.

**Figure 5:**
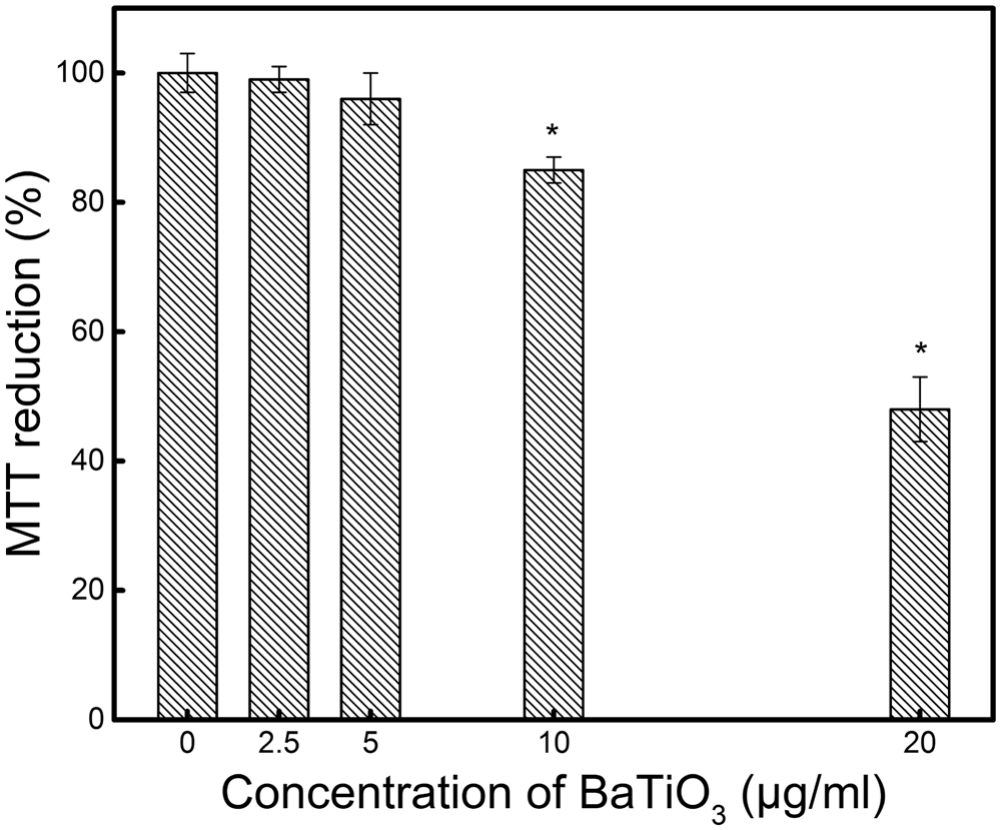
Effect of PLL coated BaTiO_3_ nanoparticles on the metabolic activity of HeLa cell as measured by MTT assay for various concentrations. Data is represented as mean ± standard error mean of three independent experiments. P < 0.05 is indicated with a*.

To verify the applicability of the two-photon photothermal microscope in live cell imaging and to check the capability of the microscope in acquiring images in the presence of scattering media, we imaged BaTiO_3_ nanoparticles internalized in a biological cell. HeLa cells are seeded in the imaging chamber and cultured for 24 hrs using standard protocol as described in the methods section. The cells are then incubated in culture media having Poly-L-Lysine (PLL) coated BaTiO_3_ nanoparticle for 6 hrs ^31^. Figure 4(a) shows the wide field microscope image of HeLa cells seeded with BaTiO_3_ nanoparticles. Figure 4(b) shows the corresponding two-photon photothermal image of the cell made by stacking different axial (Z) sections. The sections are taken after moving the microscope objective 2 µm at each step. The sections are combined by ImageJ software to make a 3D image. The figure confirms that the two-photon photothermal microscope can indeed provide high quality images of live cells. In order to check the cell viability after addition of BaTiO_3_ nanoparticle, a cytotoxicity measurements using (3-(4,5- dimethylthiazole-2-yl)-2,5-diphenyl tetrazolium bromide) assay was carried out. Figure 5 depicts the results of MTT assay performed in HeLa cells treated with PLL coated BaTiO_3_ nanoparticles having a size of 20 nm. No significant decrease in MTT reduction is observed up to 5 µg/ml of PLL coated BaTiO_3_ nanoparticle concentration. While a significant decrease in MTT reduction of 16% (p < 0.05) is observed for 10 µg/ml concentration. A sharp decrease in MTT reduction is found (p < 0.01) for 20 µg/ml of BaTiO_3_ nanoparticle treated cells.

## Conclusion

In summary a new nonlinear optical microscopic technique based on two-photon absorption induced photothermal effect capable of detecting individual nonfluorescent nanoparticles is developed. The two-photon photothermal microscope makes use of high repetition rate, near infrared laser pulses of picojule energy as the heating beam in a pump-probe measurement scheme. The microscope is successfully employed to acquire images of BaTiO_3_ nanoparticles in the size range of 20 nm to 70 nm with high sensitivity. BaTiO_3_ is a highly photostable and biocompatible material having femtosecond response time and could provide a potential label for imaging. The near infrared excitation wavelengths employed by the microscope are less toxic to living cells. Further it provides a larger penetration depth and high SNR in imaging due to lower scattering. The microscope is inherently confocal making it a potential alternative tool for three dimensional imaging in a scattering environment. The applicability of this technique in biology is demonstrated by imaging BaTiO_3_ nanoparticles internalized in HeLa cells. Detection of nanometer sized single particles using photothermal microscopy incorporated with two-photon absorption has promising applications in deep tissue imaging in biological research and clinical diagnostics. Two-photon absorption being a third order process, the technique can be employed to detect and characterize nanoparticles regardless of its symmetry.

## Methods

In this experiment we use two sets of particles to demonstrate two-photon photothermal imaging 1) commercially purchased (Sigma-aldrich, Product No. 467634) BaTiO_3_ nanoparticle having cubic structure and an average size of 70 nm and 2) BaTiO_3_ nanoparticles having an average size of 20 nm synthesized in the laboratory using sol-gel technique^38^. The latter particles have a tetragonal crystal structure as verified by X-ray diffraction technique. The samples for microscopic studies are prepared from a dilute solution of BaTiO_3_ nanopowder in ethanol. 5 mg of BaTiO_3_ powder is added to 15 ml of ethanol and sonicated for 30 minutes to form a clear solution. This solution is filtered using a 220 nm filter and spin coated on a 22 × 22 mm cleaned microscopic coverslip at 4000 rpm for 30 seconds. The spinning speed and concentration of BaTiO_3_ nanoparticles is optimized in such a way that a uniform thin layer of well isolated nanoparticles are formed. Water is added on top of the prepared coverslip before taking the images.

**Figure 6:**
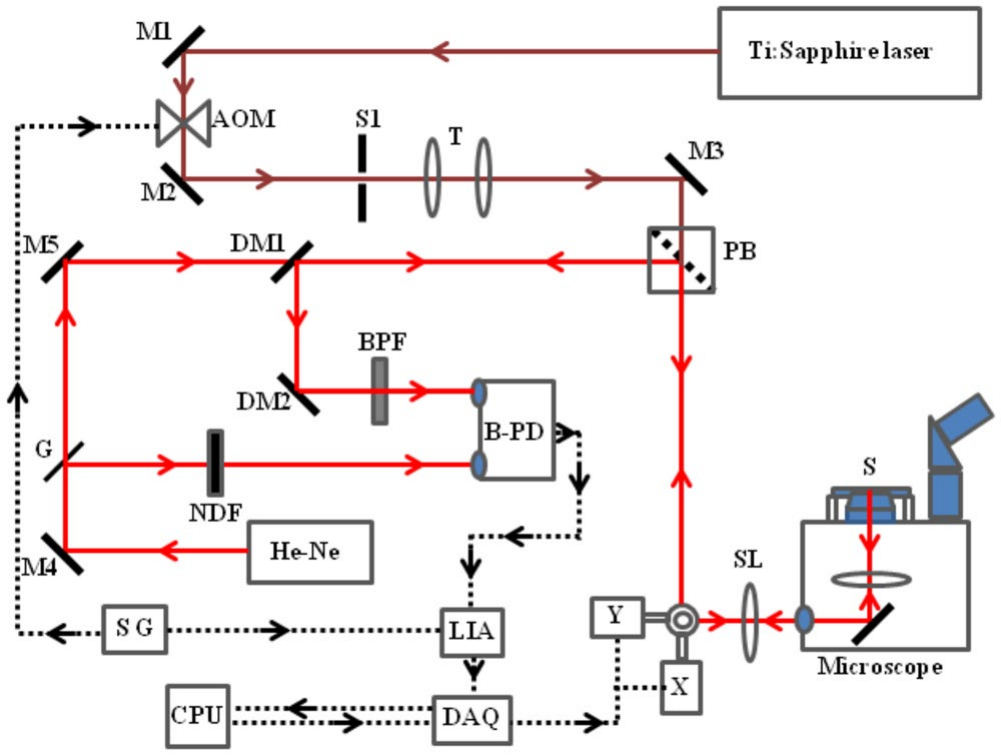
Schematic of laser scanning two-photon photothermal microscope. M: Mirror, AOM: Acousto Optic Modulator, S1: Aperture, T: Telescope, PB: Polarizing Beam splitter, X&Y: Scan Mirrors, SL: Scan Lens, S: Sample, G: Glass slide, NDF: Neutral Density Filter, B-PD: Balanced Photo Receiver, BPF: Band Pass Filter, DM: Dichroic Mirror, DAQ: Data Acquisition card, CPU: Computer, LIA: Lock-In Amplifier, SG: Signal Generator. Continuous line shows the optical path and dotted line represents the electrical signal path.

Figure 6 shows the optical layout of two-photon photothermal microscope. Here we have used a near infrared ~120 fs mode-locked Ti: sapphire laser (Mira 900, Coherent) operating at 76 MHz as the pump laser to excite the sample through two-photon absorption. The wavelength of the laser is tuned to 710 nm to coincide with the two-photon absorption band of BaTiO_3_. A 632 nm He-Ne laser (Thorlabs, HNL150L) is used as the probe beam. The pump beam is modulated at 115 kHz by an acousto optic modulator (AOM, 1205C-1, Isomet). A polarizing beam splitter combines both pump and probe beam and directs it towards the scan mirrors. The scan lens kept in front of the scan mirrors focuses the pump and probe beam to the conjugate focal plane of an inverted microscope (IX 71, Olympus, 60X, NA 1.25 objective) on which the samples as prepared above is mounted. Back scattered probe beam from the sample that returns along the same pathway is directed to a large area balanced photo receiver (2307-M, New Focus) by the polarizing beam splitter and two dichroic mirrors (DM1 and DM2). Back scattered pump beam is further filtered off by keeping an interference band pass filter (632 ± 10 nm, CVI Melles Griot) in front of the detector. The output of the photo receiver is given to a 200 MHz dual phase lock-in amplifier (SR-844, Stanford Research Systems). A two dimensional image is obtained by raster scanning the laser beam over the sample with the help of a galvanometric scanner (6215 H, Cambridge Technology Enterprises). The three dimensional sectioning is achieved by moving the microscope objective along the Z-direction. A data acquisition card (NI USB-6251) along with National Instrument LabVIEW software is used for data acquisition as well as for controlling the scan mirrors.

A stable dispersion of BaTiO_3_ nanoparticles is prepared in Poly L Lysine (PLL) with Phosphate Buffered Saline (PBS)^31^. 1 mg of BaTiO_3_ is added in to 1 ml of 0.1% PLL in PBS solution and sonicated for 12 hrs resulting 1 mg/ml PLL coated BaTiO_3_ nanoparticle. The applicability of two-photon photothermal microscopy in bioimaging is demonstrated by imaging PLL coated BaTiO_3_ nanoparticle internalized HeLa cells. HeLa cells were cultured in Dulbecco’s Modified Eagle’s Medium (DMEM) with 10% Fetal Bovine Serum (FBS) and 1% pencillin streptomycine at 37 °C in 5% CO_2_. For nanoparticle internalization study ~20,000 cells were seeded in imaging chamber and incubated for 24 hrs. After incubation for 24 hrs at 37 °C in 5% CO_2_, culture media is removed and cells were carefully rinsed with PBS solution (pH 7.4). Then a colloidal solution of BaTiO_3_ nanoparticles in culture media (5 µg/mL) was added to the imaging chamber and incubated for next 6 hrs. Two-photon photothermal imaging is carried out after 6 hrs of incubation time. The metabolic activity of the BaTiO_3_ nanoparticle treated cells are studied by MTT (3-(4,5- dimethylthiazole-2-yl)-2,5-diphenyl tetrazolium bromide) assay. For MTT assay 20,000 cells were seeded in the 96 well plate and incubated for 24 hrs. After 24 hrs of incubation, cells were treated with modified culture media containing various concentrations of BaTiO3 nanoparticle (2.5 µg/ml, 5 µg/ml, 10 µg/ml, 20 µg/ml) and incubated for another 24 hrs. Afterwards cells were incubated with MTT having concentration of 0.5 mg/ml for 4 hrs. Once MTT reduction is done supernatant is aspirated the followed by treating the sample with 100 µl of dimethyl sulphoxide (DMSO). The absorbance was measured at a wavelength of 570 nm using a 96 well plate micro plate reader. In all the experiments, control test was done on untreated cells. All the experiments were done in triplicate and three independent tests were conducted in different days. Statistical analysis of MTT assay was done by analysis of variance (ANOVA) followed by Student’s t test by setting p < 0.05 as significant and marked with asterisk in the graph. The results are reported as mean ± standard error of the mean (SEM). Cell viability after NPs incubation and after irradiation with pump powers up to 10 mW was tested by trypan blue exclusion method. The cells remain viable at these power levels, no visible damage or entry of the dye into the cytoplasm is observed. The pump energy employed in the imaging reported here is only 0.03 Nano joules (3 mW). We have repeatedly imaged the same set of cells 10–15 times to check whether the cells are getting affected. Even after repeated imaging, the cells remain intact and provide reproducible images.

## References

[1] Matsumoto, B. *Cell Biological Applications of Confocal Microscopy*. (Elsevier Science, 2003).

[2] Pawley, J. B. *Handbook of Biological Confocal Microscopy*. (Springer, 1995).

[3] Lichtman JW, Conchello JA (2005). Fluorescence microscopy. Nat Meth.

[4] Nirmal M (1996). Fluorescence intermittency in single cadmium selenide nanocrystals. Nature.

[5] Michalet X (2005). Quantum Dots for Live Cells, in Vivo Imaging, and Diagnostics. Science.

[6] Zumbusch A, Holtom GR, Xie XS (1999). Three-Dimensional Vibrational Imaging by Coherent Anti-Stokes Raman Scattering. Phys Rev Lett.

[7] Freudiger CW (2008). Label-Free Biomedical Imaging with High Sensitivity by Stimulated Raman Scattering Microscopy. Science.

[8] Nandakumar P, Kovalev A, Volkmer A (2009). Vibrational imaging based on stimulated Raman scattering microscopy. New J. Phys..

[9] Smith B (2013). Portable, miniaturized, fibre delivered, multimodal CARS exoscope. Opt Express.

[10] Boyer D, Tamarat P, Maali A, Lounis B, Orrit M (2002). Photothermal Imaging of Nanometer-Sized Metal Particles Among Scatterers. Science.

[11] Zharov V, Lapotko D (2003). Photothermal sensing of nanoscale targets. Rev. Sci. Instrum.

[12] Gaiduk A, Yorulmaz M, Ruijgrok PV, Orrit M (2010). Room-Temperature Detection of a Single Molecule’s Absorption by Photothermal Contrast. Science.

[13] Berciaud S, Cognet L, Blab GA, Lounis B (2004). Photothermal Heterodyne Imaging of Individual Nonfluorescent Nanoclusters and Nanocrystals. Phys Rev Lett.

[14] Selmke M, Cichos F (2013). Photothermal Single Particle Rutherford Scattering Microscopy. Phys Rev Lett.

[15] Berciaud, S., Cognet, L., Blab, G. A. & Lounis, B. Photothermal heterodyne imaging of individual nonfluorescent nanoclusters and nanocrystals. *Phys. Rev. Lett*. **93** (2004). 10.1103/PhysRevLett.93.257402 15697940

[16] Arunkarthick S, Bijeesh MM, Varier GK, Kowshik M, Nandakumar P (2014). Laser scanning photothermal microscopy: fast detection and imaging of gold nanoparticles. J. Microsc..

[17] Huang X, El-Sayed IH, Qian W, El-Sayed MA (2006). Cancer Cell Imaging and Photothermal Therapy in the Near-Infrared Region by Using Gold Nanorods. J. Am. Chem. Soc..

[18] El-Sayed IH, Huang X, El-Sayed MA (2006). Selective laser photo-thermal therapy of epithelial carcinoma using anti-EGFR antibody conjugated gold nanoparticles. Cancer Lett..

[19] Tong L, Wei Q, Wei A, Cheng J-X (2009). Gold Nanorods as Contrast Agents for Biological Imaging: Optical Properties, Surface Conjugation and Photothermal Effects†. Photochem. Photobiol.

[20] Tong L (2007). Gold Nanorods Mediate Tumor Cell Death by Compromising Membrane Integrity. Adv. Mater..

[21] Helmchen F, Denk W (2005). Deep tissue two-photon microscopy. Nat Meth.

[22] Two-Photon Excitation Fluorescence Microscopy. *Annu. Rev. Biomed. Eng*. **2**, 399–429 (2000). 10.1146/annurev.bioeng.2.1.399 11701518

[23] Hoover EE, Squier JA (2013). Advances in multiphoton microscopy technology. Nat Photon.

[24] Abeyasinghe N (2016). Enhanced Emission from Single Isolated Gold Quantum Dots Investigated Using Two-Photon-Excited Fluorescence Near-Field Scanning Optical Microscopy. J. Am. Chem. Soc..

[25] Lu, S., Min, W., Chong, S., Holtom, G. R. & Xie, X. S. Label-free imaging of heme proteins with two-photon excited photothermal lens microscopy. *Appl. Phys. Lett*. **96** (2010).

[26] Moger J (2012). Imaging cortical vasculature with stimulated Raman scattering and two-photon photothermal lensing microscopy. J. Raman Spectrosc..

[27] Boyd, R. W. *Nonlinear Optics*. (Elsevier Science, 2003).

[28] Mary KP (2014). Photothermal Therapy Using Gold Nanorods and Near-Infrared Light in a Murine Melanoma Model Increases Survival and Decreases Tumor Volume. J. Nanomater..

[29] Berciaud S, Lasne D, Blab GA, Cognet L, Lounis B (2006). Photothermal heterodyne imaging of individual metallic nanoparticles: Theory versus experiment. Phys Rev B.

[30] Chu, B. *Laser Light Scattering*. (Academic Press, 1974).

[31] Ciofani G (2010). Preparation of stable dispersion of barium titanate nanoparticles: Potential applications in biomedicine. Colloids Surf. B Biointerfaces.

[32] Hsieh C-L, Grange R, Pu Y, Psaltis D (2010). Bioconjugation of barium titanate nanocrystals with immunoglobulin G antibody for second harmonic radiation imaging probes. Biomaterials.

[33] Hsieh C-L, Grange R, Pu Y, Psaltis D (2009). Three-dimensional harmonic holographic microcopy using nanoparticles as probes for cell imaging. Opt Express.

[34] O’Brien S, Brus L, Murray CB (2001). Synthesis of Monodisperse Nanoparticles of Barium Titanate: Toward a Generalized Strategy of Oxide Nanoparticle Synthesis. J. Am. Chem. Soc..

[35] Gläsel H-J (1999). Preparation of barium titanate ultrafine powders from a monomeric metallo-organic precursor by combined solid-state polymerisation and pyrolysis. J. Mater. Sci.

[36] Staedler D (2012). Harmonic Nanocrystals for Biolabeling: A Survey of Optical Properties and Biocompatibility. ACS Nano.

[37] Ĉulić-Viskota J, Dempsey WP, Fraser SE, Pantazis P (2012). Surface functionalization of barium titanate SHG nanoprobes for in vivo imaging in zebrafish. Nat Protoc..

[38] Bijeesh, M. M., Nandakumar, P. & Varier, G. K. *To be publ*.

